# Mirza Kazem Mahallati (1832-1896), the Pioneer of Modern Chemistry and Pharmacy in Iran

**DOI:** 10.34172/aim.34388

**Published:** 2025-08-01

**Authors:** Ali Emadzadeh

**Affiliations:** ^1^Department of Internal Medicine, MMS.C., Islamic Azad University, Mashhad, Iran

**Keywords:** Chemistry, Pharmacy, Iran

## Abstract

Mirza Kazem Mahallati (Mirza Kazem Shimi) was one of the first graduates of Dar Al-fonun Academy who went on to become the founder of modern pharmacy and chemistry in Iran. This article looks at Mirza Kazem’s biography and his efforts in foundation of modern pharmacy in Iran.

 Born in 1832 in Mahallat, center of Iran, Mirza Kazem was a member of a family acquainted with chemistry and pharmacy.^1^ His brother, Naseer (Naseer Al –Atebba`), was a chemist and managed his pharmacy in Tehran, the capital of Iran.^1^ It is alleged that his father, Mirza Ahmad, was a descendant of Khajeh Naseer Al-din Toussi, the famous Iranian scientist in the 13^th^ Century AD.^2^ When he was a 5-year-old child, he moved with his father to Atabaat (Shia Muslims’ religious cities, at that time located in the Ottoman Empire, now in Iraq).^2^ After passing his primary traditional and religious training courses in Atabaat, he moved to Tehran.^2^ He entered Dār Al-Fonun Academy in the early 1850s.^1,2^ Dār Al-Fonun Academy was an Institute founded in Tehran in 1851 by Mīrzā Ṭāqī Khan Amīr Kabīr, the grand vizier of Nassereddin Shah Qajar, which marks the beginning of modern education in Iran.^3^ In Dār Al-Fonun, not only he learned the French language, but also the sciences, including physics, chemistry, biology, and geology.^1,2^ He graduated from that academy in 1859 and in the same year, he was sent to France with some other graduates from Dar Al-Fonun to continue their education.^1,2^ He entered the university in Rouen, France.^4^ The progression of Mirza Kazem in his studies was so impressive, as Jules Thieury, the French head of Iranian students in France, admired Mirza Kazem as a talented student, in his letter to the Persian Ambassador, Hassan Ali Khan Amir Nezaam (Figure 1).^1,2,5^ During his university time in France, he also taught the French Language to his classmates.^6^ After graduating from the university, he returned to Iran and became one of the teachers at Dār Al-Fonun Academy in 1862 (Figure 2).^1,2,6^ He officially replaced Monsieur Fochetti, an Italian pharmacist, as the professor of chemistry and pharmacy in 1870 in Dar Al-fonun.^7^ It is alleged that Monsieur Fochetti trifled in teaching students, unlike Mirza Kazem, with his unique teaching style that attracted students very well.^1^ The expertise of Mirza Kazem in chemistry, as the cornerstone of pharmacy, was the main reason for his success in teaching pharmacy.^1^ The reputation of Mirza Kazem Mahallati as a distinguished and expert professor in chemistry and pharmacy attracted Iranian and even foreign authorities.^1^ Etemaad Al-saltaneh, one of the famous and impressive Qajar court men in the era of Nassereddin Shah Qajar, praised Mirza Kazem as “*kholaase ye a’saar va yegaane ye advaar*” meaning “the extract of centuries and times”.^8^ For his efforts in development of pharmacy and chemistry in Iran, he was honored to receive impressive awards, among which, the 2nd degree Lion and Sun medal from the Iranian Royal Court, the Russian St. Stanislas medal,^1,8^ and the Order of Academic Palms (Ordre des Palmes Académiques) by Poincaré, Minister of Education of France, are noticeable.^1,9,10^ Mirza Kazem also compiled many textbooks, most of them in the field of pharmacy and chemistry, in Persian.^1^ Some of his books are as follows^1^:

- The Principles of Organic Chemistry
- The Principles of Modern Chemistry
- *Tazkera Al Advieh* Nasseri (including three volumes, in pharmacy and also in translation)^1,5^
- *Akkaasi* (Photography), including five parts (an introduction, three chapters, and a conclusion part), about physical and chemical terms used in photography, the structure of a photo camera, and the methods of photo taking by a camera.^5^

**Figure 1:**
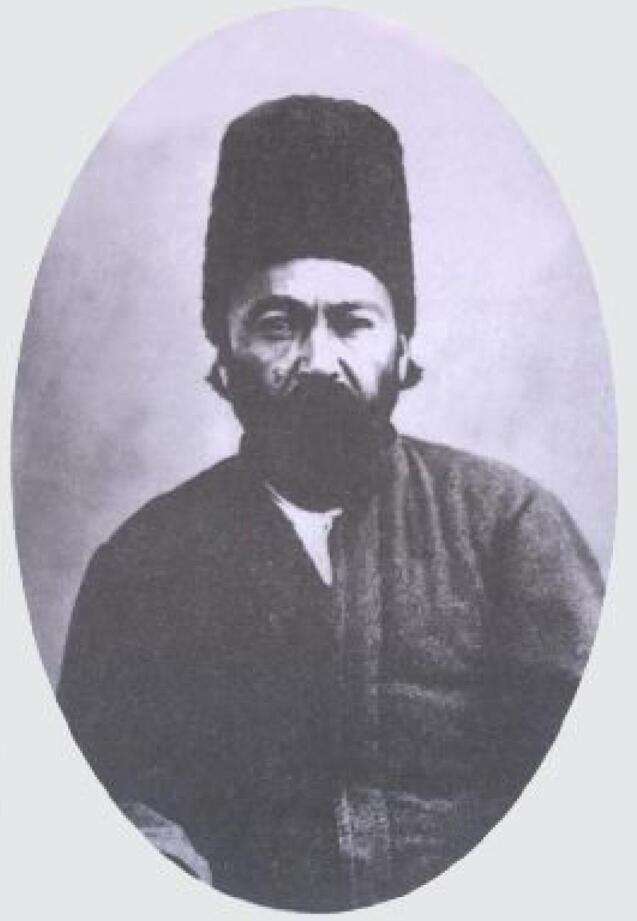
Mirza Kazem Mahallati (Mirza Kazem Shimi), (Adapted from Motamedi with permission).^5^

**Figure 2:**
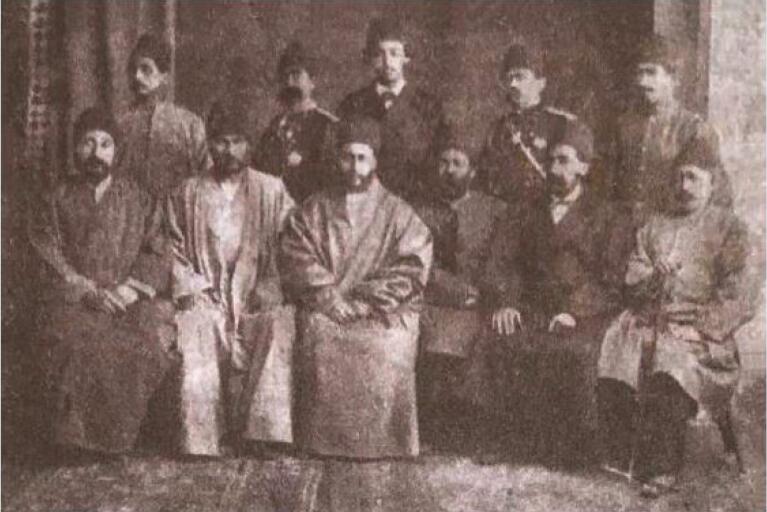
Academic Staff of Dar Alfonun, Mirza Kazem Mahallati (seated: 3^rd^ person from the right), (Adapted from Motamedi with permission).^5^

 He also compiled some other books in the fields of chemistry, physics, and pharmacy.^6,11^

 Mirza Kazem Mahallati knew Arabic and French languages, as well as his mother language, Persian,^11^ so he translated some historical books from French to the Persian language, mostly ruled by Nassereddin Shah. A number of them are as follows^12^:

- The War between Germany and France
- The War between Ottoman Turkey and Russia
- The history of war in the East (in 2 volumes)^5^
- Why/Because (in Persian: “*cheraa, be in jehat*”): a scientific book translated by Mirza Kazem Mahallati and Mohammad Ali Foroughi. In its original form, this book consisted of the questions of two children about scientific problems with simple answers to their questions. In the Persian translation, Mirza Kazem Mahallati inserted the names of his sons, Ahmad and Mahmood, instead of the names of French children, and his own name, Kazem, as the person who answers the children’s questions.^5,11^ These questions and answers included topics like the weather, the earth, gravity, and electricity (Figure 3).^5^

**Figure 3:**
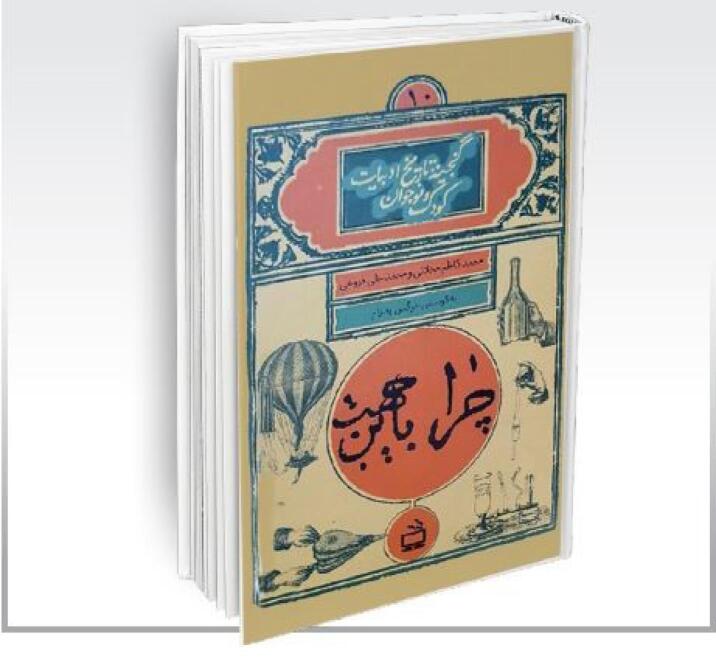
Why/Because (in Persian: *cheraa, be in jehat*): A Scientific Book Translated by Mirza Kazem and Mohammad Ali Foroughi. In its original form, this book consisted the questions of two children about scientific problems with simple answers to their questions. In the Persian translation, Mirza Kazem Mahallati inserted the names of his own sons, Ahmad and Mahmood, instead of the names of French children, and his own name, Kazem, as the person who replies to the children’s questions.^5,11^ These questions and answers included topics like the wind, the weather, the earth, gravity, and electricity.^5^ (Adapted from Motamedi with permission).^5^

 With his impressive attempts at translation, he put himself as one of the most prominent translators in the second half of the 19^th^ century in Iran.^12^

 He also wrote numerous scientific articles. For instance, he wrote many papers in an academic journal called “*Rooznaame ye Daanesh*” which means “scientific newspaper” (in Persian). This journal was published every two weeks and spread among Dār ul-Funun students freely.^1,13^ He was the managing editor of that journal.^5,13^ It was substituted for the previous journal of Dar Al-fonoun, “*Roozname ye Elmi*”.^5^ With a glance at Mirza Kazem’s articles in these journals, it is understood that he was an updated person with good access to new academic articles and journals published in European countries.^1^

 He also enrolled as the manager of all laboratories of Dār Al-Fonun Academy.^1^ His expertise in chemistry was remarkable, as he made devices for producing sulfuric acid and also made a small factory for processing sugar with limited facilities at that time at Dār Al-Fonun.^11^ He was also involved in discovering mines and processing the minerals by Nassereddin Shah Qajar’s order.^11^ His efforts in promoting pharmacy, chemistry, medicine, and mineralogy in Iran attracted the attention of the royal court, so Nassereddin Shah noticed Mirza Kazem and supported him.^11^ For his deep involvement in chemistry, he was nicknamed by his colleagues and students as “Mirza Kazem Shimi” (“Shimi” meaning “Chemistry” in the Persian language), as it became a heritage in his family as mentioned below. As he was an expert chemist and spent much time in his chemical laboratory, he became known by his colleagues as “Lavoisier of Persia”, in memory of the distinguished French chemist, Antoine-Laurent de Lavoisier.^11^

 Other than his efforts in the progression of pharmacy and chemistry at Dār Al-Fonun Academy and the translation of historical books, he also had a significant position in health policy-making in Iran. In Nassereddin Shah Qajar’s period, a council named “*Majles-e Hefz al- Sehheh*,” meaning Health Council, was founded for managing health problems in Iran.^14,15^ Its first president was Dr. Joseph Desire Tholozan, the special physician of Nassereddin Shah.^14^ This council comprised Iranian and European doctors and some official government members.^15^ One of those members was Mirza Kazem Shimi (Figure 4).^15,16^

**Figure 4:**
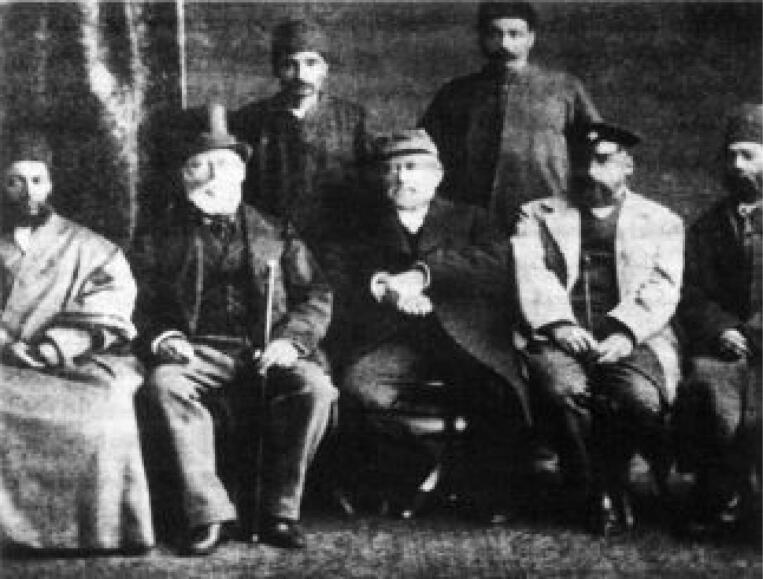
Members of the Sanitary Council (Majles-e Hefz al-Sehheh), unknown date, probably before 1895. From left to right: Dr. Mirza Ali Rais al-Atebba, Dr. Dickson, Mirza Abdullah Tabib, Dr. Joseph Desire Tholozan, Dr. Ali Akbar Khan-e Nafisi, Dr. Cherebinin, and Mirza Kazem Shimi. (Adapted from Azizi with permission).^15^ The main source: Ebrahimnejad H. Medicine, public health, and the Qajar state: patterns of medical modernization in nineteenth-century Iran Leiden-Boston: Brill Publication; 2004.^16^

 Mirza Kazem Shimi was also an expert in the field of Persian Literature, and composed elaborate Persian poems.^2^

## Personal Life

 Mirza Kazem Shimi married Fatemeh (Yaaghieh Beygom Sultan), Lotf Ali Khan Sardar’s daughter.^11^ He made much effort to educate his two sons, Ahmad and Mahmood, and his one daughter, Maryam Sultan (Havva).^11^ His wife died 14 years before him.^11^ His wife’s death affected him so much as he wrote an impressive poem in her memory.^11^

 His colleagues and students mentioned Mirza Kazem as a humble and hardworking, man who was kind to all people.^11^

## Aftermath

 He passed away in Tehran on Farvardin 17, 1275 Solar Hijri (Shawwal 21, 1313 Lunar Hijri, April 5 1896 AD).^11^ He was buried in Ibn Babawayh Cemetery in Shahr-e Ray (City of Rey), south of Tehran.^2,11^

## Legacy

 When having a family name was mandatory in Iran, Mirza Kazem Shimi’s children adopted the family name “Shimi” in honor of their father.^5^ Some of his children were involved in chemistry and pharmacy like him; among them, Dr. Mahmoud Shimi was so famous and became one of the impressive teachers of Dār Al-Fonun Academy, like his father.^11,17^

**Figure 5:**
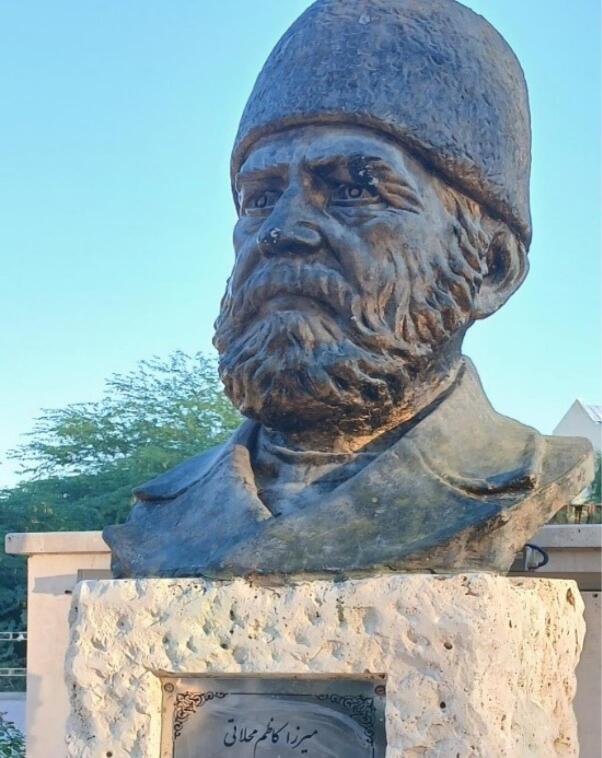
Statue of Mirza Kazem Mahallati, Kish Island, Iran, Jan. 2025. Photo taken by the author.

 In Kish Island, Iran, the statue of Mirza Kazem Mahallati was unveiled in 1999 in honor of his valuable attempts in developing pharmacy and chemistry in Iran (Figure 5).

 The medical sciences and chemistry communities of Iran always admire Mirza Kazem Shimi for his unforgettable efforts, but some of the aspects of his life are obscure; for instance, there is no copy or manuscript of some of his books like “*Tazkera Al Advieh Nasseri*”.^1^ Also, a little is known about his university time in France, so more research about Mirza Kazem Mahallati is suggested.
